# Recognition of dipole-induced electric field in 2D materials for surface-enhanced Raman scattering

**DOI:** 10.3389/fchem.2023.1183381

**Published:** 2023-04-07

**Authors:** Yuxue Yang, Shen Ao, Jiaqi Wang, Wangyang Fu, Xiangxuan Liu, Weipeng Wang

**Affiliations:** ^1^ High-Tech Institute of Xi’an, Xi’an, Shaanxi, China; ^2^ Key Laboratory of Advanced Materials (MOE), School of Materials Science and Engineering, Tsinghua University, Beijing, China

**Keywords:** two-dimensional (2D) materials, chemical enhancement, surface enhancement Raman scattering, dipole interaction, vibration selectivity

## Abstract

The application of two-dimensional (2D) materials, including metallic graphene, semiconducting transition metal dichalcogenides, and insulating hexagonal boron nitride (h-BN) for surface-enhancement Raman spectroscopy has attracted extensive research interest. This article provides a critical overview of the recent developments in surface-enhanced Raman spectroscopy using 2D materials. By re-examining the relationship between the lattice structure and Raman enhancement characteristics, including vibration selectivity and thickness dependence, we highlight the important role of dipoles in the chemical enhancement of 2D materials.

## 1 Introduction

During the past few decades, the increasing demand for environmental monitoring, food safety, and rapid diagnosis has triggered extensive research in the development of biochemical sensor devices with high sensitivity and selectivity. Raman spectroscopy has been used as an important molecular analytical tool since Raman and Krisnan discovered it in 1928 (Raman and Krishnan, 1928). Raman spectroscopy stands out for its excellent performance, including non-destructive, label-free, rapid sensing, and the ability to recognize the “fingerprint” of the target molecule’s vibration. However, in general, only one of one million incident photons can undergo Raman scattering. Such an extremely low probability of Raman scattering leads to a weak Raman intensity, which hinders the application of Raman spectroscopy to trace detection. In 1974, Fleischman discovered the surface-enhanced Raman scattering (SERS) effect (Fleischmann et al., 1974), which provides a method for biochemical detection even down to the molecular level (Zhang Y. Q. et al., 2017; Wei et al., 2018; Gu et al., 2023) on the basis of two widely accepted enhancement mechanisms: the electromagnetic mechanism (EM) and chemical mechanism (CM). On the basis of the EM, noble metals with plasmonic properties have been widely adopted as highly sensitive SERS substrates (Dasary et al., 2009; Zhou et al., 2010; Yang et al., 2014; Liu et al., 2018). Although CM involves more complex interactions, it has been widely studied to understand the chemical interactions between the analyte and substrate, taking two-dimensional (2D) materials as ideal platforms (Ling et al., 2010; Ling et al., 2014; Park and Jhon, 2019).

Inspired by the study of the chemical enhancement mechanism in metal-based SERS (Sanchez-Cortes and Garcia-Ramos, 2000; Shibamoto et al., 2007; Gruenke et al., 2016), researchers have adopted the chemical enhancement mechanism based on charge transfer to explain the enhancement of Raman scattering in 2D materials. However, experimental evidence for enhanced Raman scattering in 2D materials suggests that the chemical enhancement effect is not solely due to charge transfer. In this article, we review the basic principles of SERS. Upon extensive re-examination of the chemical enhancement phenomenon in 2D materials, we emphasize the important contribution of locally induced electric fields on the material surface by dipoles. Although this effect enables Raman enhancement through local electric fields, it is classified as chemical enhancement, because it stands for one type of substrate-analyte interaction, distinguishing it from surface plasmon-induced electromagnetic enhancement.

## 2 The mechanisms of SERS of 2D materials

Raman scattering is the inelastic collision of photons caused by molecules during their interaction. After absorbing the incident photon, the molecule transitions to a virtual energy level and releases the scattered photon when it returns to its molecular level. In this process, photons generating the electromagnetic field can gain or lose energy from the molecules (related to the difference between the energy levels of the molecule before and after scattering), resulting in a change in the frequency (or energy) of the scattered photons (Cheng and Xie, 2004; Evans and Xie, 2008). Therefore, the energy level information of a molecule can be obtained using Raman spectroscopy for fingerprint identification (Ferrari, 2007; Zhang et al., 2013).

Raman scattering is sensitive to changes in molecular polarization. When the molecule interacts with the electromagnetic field from the incident photon, the dipole moment is induced, and the photon is scattered; the relationship between the dipole moment, electromagnetic field, and polarizability of the molecule can be represented as (Moskovits, 2005),

$$\mu_{ind}=E_{inc}\cdot \alpha_{m}$$

where *μ*_ind_ is the induced dipole moment, *E*_inc_ is the incident electromagnetic field, and *α*_m_ is the polarizability of the molecule. By taking the vibrational mode of molecules as a simple harmonic vibrational model, the scattering process can be represented as

$$\mu_{ind}=\alpha_{m}E_{inc}\cos\left(2\pi v_{inc}t\right)+{\left.\frac{\partial \alpha }{\partial q}\right|}_{q_{0}}\frac{q_{0}E_{inc}}{2}\left\{\cos\left[2\pi \left(v_{inc}-v_{vib}\right)t\right]+\cos\left[2\pi \left(v_{inc}+v_{vib}\right)t\right]\right\}$$

where *v*_inc_ is the frequency of the incident photon and *v*_vib_ is the frequency of the vibration mode. The second part of the equation describes Raman scattering; the intensity of Raman scattering mainly depends on the intensity of the incident electromagnetic field and the change in molecular polarizability, corresponding to the EM and CM mechanisms of SERS (Jones et al., 2019). In the following discussion, we review the two basic mechanisms in detail.

### 2.1 Electromagnetic field enhancement

**FIGURE 1:**
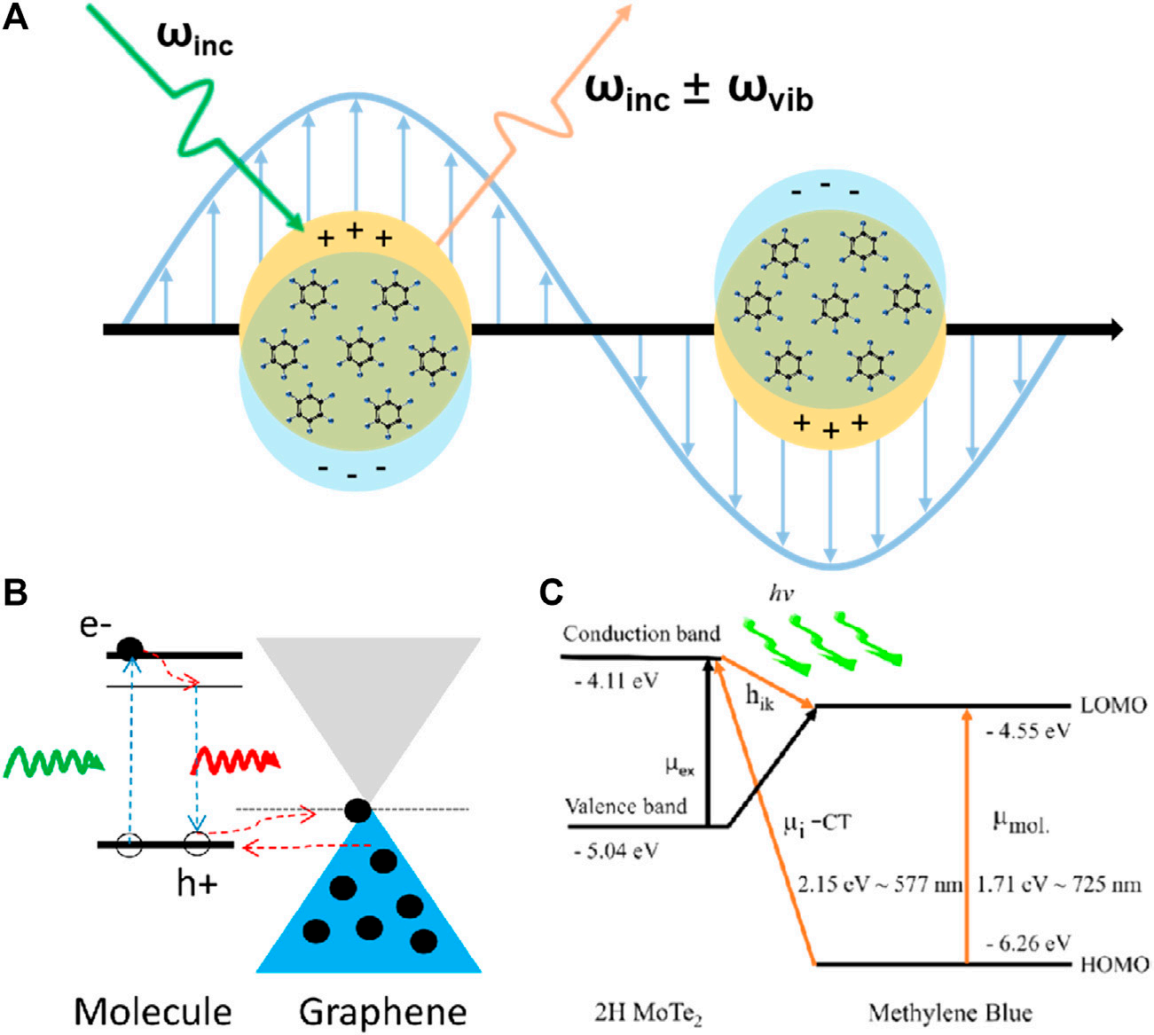
**(A)** Schematic of EM on a metal SERS substrate. **(B)** Energy-level diagram showing the GSCT process in GERS. **(C)** Energy-level diagram showing the PICT process between a 2D MoTe_2_ film and methylene blue molecule.

EM results from the enhancement of the local electromagnetic field induced by local surface plasmon resonance (LSPR) and contributes to the major part of the enhancement factor (EF) for SERS (Willets and Van Duyne, 2007; Sepulveda et al., 2009), shown in Figure 1A. In this mechanism, the incident and scattered electromagnetic fields are both enhanced by the LSPR of the nanostructure. When the incident electromagnetic field interacts with materials possessing a dielectric constant with a large negative real part and a small positive imaginary part, the oscillating electric field excites the free electrons for reciprocating motion. When the oscillating electrons resonate with the incident electromagnetic field, the absorption capacity of the nanostructures for the incident electromagnetic field reaches its peak, and Raman spectroscopy obtains the strongest electromagnetic enhancement.

The LSPR-based enhancement can be deduced by considering the driven harmonic oscillator model (Schlucker, 2014). The incident electromagnetic field *E*_inc_ (*v*_inc_) generates an induced electromagnetic field *E*′_inc_ (*v*_inc_) in the nanostructure. The primary electric field *E*_l_ (*v*_inc_) can be represented as

$$E_{l}\left(v_{inc}\right)=E_{inc}\left(v_{inc}\right)+E_{inc}^{\prime }\left(v_{inc}\right)=f\left(v_{inc}\right)E_{inc}\left(v_{inc}\right)$$

The molecule is excited by a primary electric field and radiates a scattering field (or secondary electric field) $E_{s}\left(v_{inc}\pm v_{vib}\right)=aE_{l}\left(v_{inc}\right)$ ; then, a secondary induced electric field *E*′_s_ (*v*_inc_ ± *v*_vib_) is generated in the nanostructure.

The secondary local electric field *E*_sl_ (*v*_inc_ ± *v*_vib_) can be represented as follows:

$$\begin{array}{l} E_{sl}\left(v_{inc}\pm v_{vib}\right)=E_{s}\left(v_{inc}\pm v_{vib}\right)+E_{s}^{\prime }\left(v_{inc}\pm v_{vib}\right)\\ =af^{\prime }\left(v_{inc}\pm v_{vib}\right)E_{s}\left(v_{inc}\pm v_{vib}\right)=af^{\prime }\left(v_{inc}\pm v_{vib}\right)f\left(v_{inc}\right)E_{inc}\left(v_{inc}\right) \end{array}$$

Hence, Raman intensity can be represented as follows:

$$I_{SERS}=a^{2}{\left|f^{\prime }\left(v_{inc}\pm v_{vib}\right)f\left(v_{inc}\right)\right|}^{2}I_{0}$$

Because *v*_vib_ << *v*_inc_, the frequencies of the incident photons and scattered photons are close to each other; thus,

$$f^{\prime }\left(v_{inc}\pm v_{vib}\right)\approx f\left(v_{inc}\right)=\left|\frac{E_{l}\left(v_{inc}\right)}{E_{inc}\left(v_{inc}\right)}\right|$$

The EF of EM can be represented as follows:

$$EF_{EM}={\left|f^{\prime }\left(v_{inc}\pm v_{vib}\right)f\left(v_{inc}\right)\right|}^{2}\approx {\left|\frac{E_{l}\left(v_{inc}\right)}{E_{inc}\left(v_{inc}\right)}\right|}^{4}$$

The SERS enhancement from the EM mechanism is equal to the fourth power of the electric field enhancement.

### 2.2 Chemical enhancement

Compared to the mature theoretical explanation of EM, the understanding of CM has been hindered until recently. Owing to the high electromagnetic enhancement effect caused by plasmons, it is difficult to directly elucidate the chemical enhancement mechanism of metal-based SERS substrates. However, some 2D materials have been found to have Raman enhancement without generating plasmons. With the interference of electromagnetic enhancement excluded, these plasmon-free 2D materials serve as excellent platforms to study the chemical enhancement mechanism to promote researchers’ understanding of the mechanism behind SERS.

The charge transfer mechanism is the most accepted chemical enhancement mechanism and is involved in most substrates, including ground-state charge transfer (GSCT) and photoinduced charge transfer (PICT) (Jensen et al., 2008), as shown in Figures 1B, C. The former is a non-resonant chemical enhancement process that describes the change in molecular electron density distribution by interactions between adsorbed molecules and the substrate under non-excitation conditions (Ling et al., 2012a). In contrast, PICT is a wavelength-dependent mechanism and provides the strongest chemical enhancement when the excitation light and arrangement of energy levels in the SERS system satisfy the energy matching relationship, that is, when resonance occurs (Adhikari et al., 2021; Wang et al., 2021; Zhao et al., 2021). According to Lombardi and Birke’s theory (Lombardi and Birke, 2009), the polarizability of molecules consists of three terms that are responsible for molecular resonance, substrate-to-molecule charge transfer, and molecule-to-substrate charge transfer.

### 2.3 Dipole-induced Raman enhancement

**FIGURE 2:**
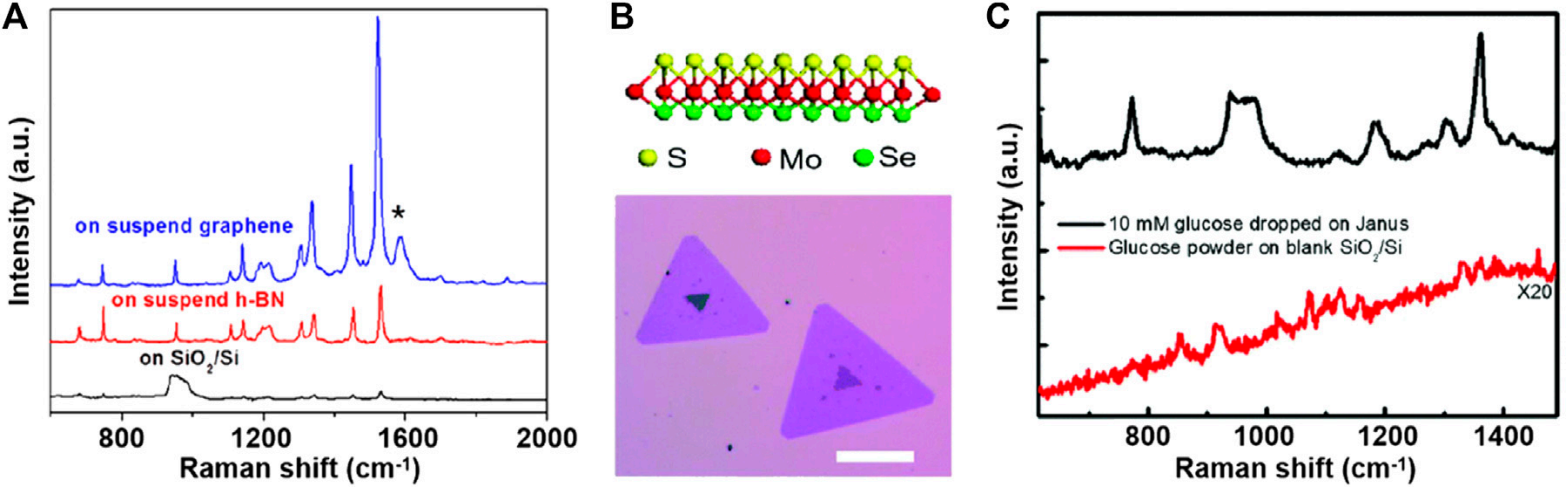
**(A)** Comparison of the Raman spectra of the 4-Å CuPc molecule on a blank SiO_2_/Si (black line), on suspended h-BN (red line), and on suspended graphene (blue line) substrates. **(B)** Schematic illustration (upper panel) and optical image (lower panel) of monolayer Janus MoSSe. **(C)** Raman spectra of solid glucose powder on the blank SiO_2_/Si substrate and 10-mM glucose solution dropped on the monolayer Janus MoSSe substrate.

However, the charge transfer mechanism fails to account for all chemical enhancement effects, such as the unforeseen Raman enhancement of hexagonal boron nitride (h-BN) and the anisotropic Raman enhancement of black phosphorus and ReS_2_ (Lin et al., 2015). Hence, we emphasize the important role of the **built-in electric field (dipole)** in 2D materials in SERS. Ling et al. (2014) deposited CuPc molecules on 2D h-BN and examined its chemical enhancement phenomenon. Because of its large band gap of more than 5.9 eV (Zhang K. L. et al., 2017), h-BN should have a negligible charge transfer capacity; however, its strong in-plane local field impacts the dipole moments of CuPc molecular vibrations. This dipole–dipole interaction mechanism exhibits a different mode-selective rule associated with the magnitude and orientation of the dipole moment of the vibration mode (Figure 2A). On graphene, the magnitude of the EF is determined by the ease of charge transfer, and the selectivity of vibrational modes depends on their frequency, i.e., energy. Conversely, on h-BN, the intralayer dipole results in the optimal enhancement of the vibrational mode of CuPc with the largest dipole moment at a frequency of 1,143 cm^−1^. In addition, the out-of-plane dipole component of 2D materials not only contributes to chemical enhancement but also is easier to manipulate. Lou et al. (Zhang J. et al., 2017; Jia et al., 2020) fabricated the single-layer MoSSe with surface Se atoms replaced by S atoms (Figures 2B, C); the continuous dipoles of S-Mo-Se generate a substantial electric field generated from the 2D material, producing a strong chemical enhancement effect. Through surface functional groups or heterostructures, dipoles could be introduced into non-polar surfaces as well, leading to superimposed chemical enhancement; details are discussed in Section 3.

According to Fermi’s golden rule, the electron transition probability *w*_lk_ can be represented as follows:

$$w_{lk}=\frac{2\pi }{\hbar }g\left(E_{k}\right){\left|H_{kl}^{\prime }\right|}^{2}$$

where *g* (*E*_k_) is the density of states and *H*′_kl_ is the matrix element for the transition between the lowest unoccupied molecular orbital (LUMO) and the highest occupied molecular orbital (HOMO). Considering graphene and h-BN, two diametrically opposed SERS substrates, the charge transfer interaction with graphene can increase the electron density of states, because graphene has numerous available electron states around the LUMO and HOMO levels. In contrast, the dipole–dipole interaction in h-BN results in a local symmetry-related perturbation and increases the matrix element. The CM for the majority of 2D materials can be considered as a mixture of the two types of interactions.

## 3 SERS by 2D materials

Although metallic nanostructures induce a strong local electric field, the alignment of the Fermi level at the metal surface and the LUMO energy level of the target molecule is generally suboptimal for CM enhancement. Furthermore, disadvantages, including poor adsorption performance, side reactions of the adsorbate, and strong fluorescent background, limit its application (LeRu and Etchegoin, 2009; Li et al., 2010; Cong et al., 2019). The discovery of graphene-based surface-enhanced Raman scattering (G-SERS) has drawn attention to 2D materials for SERS. To date, diverse 2D materials have been adopted to study the Raman enhancement on different 2D material surfaces, such as graphene, h-BN, transition metal dichalcogenides (TMDs), metal oxides, and black phosphorous.

### 3.1 Graphene

Graphene is a unique solid-state material because all sp2 carbon atoms are located on the surface in a honeycomb-like form and are extremely sensitive to environmental changes, making graphene suitable for sensing applications. This structure endows graphene with a chemically inert surface, high π-electron density at the surface, optical transparency, flexibility, and mechanical strength, providing a good material foundation for graphene-enhanced Raman scattering (GERS) research.

Graphene is a typical chemical enhancement 2D material that mainly depends on charge transfer interactions because of its zero-band gap and structure composed of the same atoms (all bonds are non-polar bonds). Research has demonstrated that a chemically similar group leads to an increased degree of charge transfer and polarizability tensor (Ling and Zhang, 2010), thus increasing the Raman scattering cross section of the target molecules. For graphene, vibrational modes involving long pairs or π electrons usually exhibit the strongest enhancement. For example, Ling et al. (2010) demonstrated that phthalocyanine (Pc) and protoporphyrin IX (PPP) exhibit a stronger Raman signal on single-layer graphene than Rhodamine-6G (R6G) and crystal violet (CV) because of their aromatic and macrocyclic structures similar to that of graphene. In addition to the different groups, different molecular orientations also affect the strength of the GSCT interaction with graphene. Heat treatment of CuPc on graphene imposes a lying-down orientation, producing a stronger enhancement by the interaction of π electrons (Ling et al., 2012b).

For 2D materials with charge transfer interaction, Raman enhancement to different vibration modes depends on the vibration frequency because a larger frequency of the vibration mode results in easier charge transfer. The number of graphene layers affects the charge-carrier distribution, thus affecting the Raman enhancement properties of graphene. Ling et al. (2013) deposited equal-density PPP or CuPc on one to six layers of graphene; different matches to the energy level of the target molecules led to differences in the Raman intensity, and monolayer graphene ensured the highest EF. In addition, graphene quenches fluorescence, and the fluorescence background can be suppressed by a factor of approximately 10^3^ in GERS (Zhao et al., 2015; Brill et al., 2021).

A series of studies has recently been conducted to enhance the dipole moments by modifying the functional groups on graphene (or graphene oxide), aiming for a more sensitive SERS performance. The incorporation of functional groups involves more molecular dipoles in the system, where the induced local electric fields trigger stronger Raman signals. Mao et al. (2014) applied a mild O_2_ plasma treatment to graphene to generate various oxygenated species, including epoxy and carbonyl groups. They confirmed the significance of the built-in local electric field extending to the absorbed molecules owing to the local dipole moments. Sung et al. (Huh et al., 2011) anchored oxygen-containing functional groups on large-scale graphene assisted by the UV-ozone oxidization technique. They attributed an enhancement factor of up to 10^4^ to the enhanced local electric field underlying the deduced dipole moment. Chiu et al. (2021) employed cation bonding to further realize a significant increase in the dipole moments of oxygen-containing functional groups on graphene. Researchers have confirmed the existence of Cu^+^ ions rather than nanoparticles, which thoroughly eliminate the typical electromagnetic and chemical mechanisms. The formed Cu-O-C bonds trigger a local electric field and polarization of the analyte molecules, suggesting a new chemical mechanism of SERS.

### 3.2 Hexagonal boron nitride

Compared to graphene, its similar structure and diverse electrical properties make h-BN another typical 2D material whose chemical enhancement depends mainly on dipole–dipole interactions. Regarding its localized in-plane electric field, the dipole mechanism in h-BN is a single-layer effect, i.e., the chemical enhancement of h-BN is independent of its thickness (Ling et al., 2014). However, the adsorption performance of h-BN depends on its thickness, resulting in stronger Raman signals in SERS substrates containing monolayer h-BN. Owing to their different chemical enhancement mechanisms, the Raman enhancements of graphene and h-BN have different vibration-mode selectivities depending on the frequency and dipole moment of the vibration mode, respectively.

The surface of h-BN is free of charge traps and dangling bonds and has high oxidation resistance; hence, atomically thin h-BN maintains stability in a variety of test environments (Cai et al., 2016; Chugh et al., 2019). Therefore, h-BN is also utilized as a protective barrier or reusable coating for metallic nanostructures.

### 3.3 Transition metal dichalcogenides

Owing to the relatively small bandgap and polar covalent bonds in the structure, the Raman enhancement of TMDs is considered to be influenced by both of the aforementioned chemical enhancement mechanisms. Although both of these interactions are relatively weak, TMDs can provide significant Raman enhancement under the combined action of both mechanisms, for example, the vibration mode at 1,531 cm^−1^ of CuPc on MoS_2_. To date, chemical Raman enhancement based on TMDs has been reported to reach electromagnetic enhancement levels (EF on the order of 10^9^–10^10^) with detection limits as low as 10^−14^ to 10^−15^ M for R6G (Tao et al., 2018).

Compared with h-BN, TMDs have dipoles in the out-of-plane direction, which offers the potential for a three-dimensional dipole-driven Raman enhancement effect. These in-layer dipoles can be modulated by introducing structural disorder (Sun et al., 2014) or other atoms. Seo et al. (2020) deposited ReO_x_S_y_ for ultrahigh SERS EF, and the O atom in the structure is believed to affect the dipole and electronic structure of two-dimensional flakes owing to its higher electronegativity. In the Janus structure mentioned previously, the entire layer of Se atoms on top of MoSe_2_ is replaced by S atoms, generating a strong dipole in the out-of-plane dipole.

Additionally, stacking different 2D materials to form heterostructures shows great potential in achieving higher SERS effects. The graphene/ReO_x_S_y_ hetrostrusture exploited the combined effects of dipole-dipole interaction and charge transfer mechanisms (Seo et al., 2020). By tuning the electronic structure of the 2D materials through stacking, it is possible to promote charge transfer between molecules and the substrate (Chen et al., 2020; Qiu et al., 2020). Furthermore, some new mechanisms on 2D heterostructures have been reported, such as plasmon on 2D materials (Ghopry et al., 2021) and non-radiative energy transfer (Dandu et al., 2020).

## 4 Conclusion and outlook

The Raman enhancement of 2D materials has attracted considerable attention in recent years owing to their stability and strong chemical enhancement. This plasmon-free SERS substrate provides a suitable platform for studying CM. In this article, the chemical enhancement of various 2D materials is reviewed briefly. In addition to the charge transfer between the substrate and analyte, the electric field induced by the dipole in 2D materials is an important source of chemical enhancement. To date, SERS substrates based on 2D materials have achieved enhanced performance comparable to that of metal-based SERS substrates by combining and modulating two types of CM, showing a promising future for ultrasensitive SERS sensing utilizing both EM and CM.

## Data Availability

The original contributions presented in the study are included in the article/Supplementary Material, further inquiries can be directed to the corresponding author.
